# Quantification of [^11^C]-*meta*-hydroxyephedrine uptake in human myocardium

**DOI:** 10.1186/s13550-014-0052-4

**Published:** 2014-09-26

**Authors:** Hendrik J Harms, Stefan de Haan, Paul Knaapen, Cornelis P Allaart, Mischa T Rijnierse, Robert C Schuit, Albert D Windhorst, Adriaan A Lammertsma, Marc C Huisman, Mark Lubberink

**Affiliations:** Department of Radiology and Nuclear Medicine, VU University Medical Center, P.O. Box 7057, 1007 MB Amsterdam, the Netherlands; Department of Cardiology, VU University Medical Center, P.O. Box 7057, 1007 MB Amsterdam, the Netherlands; Section of Nuclear Medicine and PET, Uppsala University, Akademiska sjukhuset, 751 85 Uppsala, Sweden

**Keywords:** Myocardial innervation, [^11^C]HED, Absolute quantification, Retention index

## Abstract

**Background:**

The aims of this study were to determine the optimal tracer kinetic model for [^11^C]-*meta-*hydroxyephedrine ([^11^C]HED) and to evaluate the performance of several simplified methods.

**Methods:**

Thirty patients underwent dynamic 60-min [^11^C]HED scans with online arterial blood sampling. Single-tissue and both reversible and irreversible two-tissue models were fitted to the data using the metabolite-corrected arterial input function. For each model, reliable fits were defined as those yielding outcome parameters with a coefficient of variation (CoV) <25%. The optimal model was determined using Akaike and Schwarz criteria and the *F*-test, together with the number of reliable fits. Simulations were performed to study accuracy and precision of each model. Finally, quantitative results obtained using a population-averaged metabolite correction were evaluated, and simplified retention index (RI) and standardized uptake value (SUV) results were compared with quantitative volume of distribution (*V*_T_) data.

**Results:**

The reversible two-tissue model was preferred in 75.8% of all segments, based on the Akaike information criterion. However, *V*_T_ derived using the single-tissue model correlated highly with that of the two-tissue model (*r*^2^ = 0.94, intraclass correlation coefficient (ICC) = 0.96) and showed higher precision (CoV of 24.6% and 89.2% for single- and two-tissue models, respectively, at 20% noise). In addition, the single-tissue model yielded reliable fits in 94.6% of all segments as compared with 77.1% for the reversible two-tissue model. A population-averaged metabolite correction could not be used in approximately 20% of the patients because of large biases in *V*_T_. RI and SUV can provide misleading results because of non-linear relationships with *V*_T_.

**Conclusions:**

Although the reversible two-tissue model provided the best fits, the single-tissue model was more robust and results obtained were similar. Therefore, the single-tissue model was preferred. RI showed a non-linear correlation with *V*_T_, and therefore, care has to be taken when using RI as a quantitative measure.

**Electronic supplementary material:**

The online version of this article (doi:10.1186/s13550-014-0052-4) contains supplementary material, which is available to authorized users.

## Background

Recently, non-invasive imaging of sympathetic innervation of the myocardium using PET [1-5] or SPECT [6-8] has gained interest based on its ability to predict life-threatening ventricular arrhythmias [9-11] and to assess whether implantable cardioverter defibrillator (ICD) therapy is appropriate [12,13]. It has been shown that sympathetic nerve terminals in the myocardium are more sensitive to ischemic damage than cardiomyocytes [9,14-21]. In addition, the area of innervation defects often exceeds the area of non-viable scar tissue [9,11,16,17]. More importantly, it has been shown that areas of viable myocardium, but with impaired innervation, are related to inducible ventricular tachycardias originating from these areas [22]. Therefore, non-invasive imaging of sympathetic innervation may play a key role in risk stratification and treatment planning in ischemic cardiomyopathy.

Sympathetic innervation can be measured using the PET tracers [^18^ F]-6-fluorodopamine [2], [^11^C] epinephrine [1,3] or [^11^C]-*meta*-hydroxyephedrine ([^11^C]HED) [4,5], with [^11^C]HED being used most often. Analysis of [^11^C]HED and [^11^C] epinephrine data often has been performed using the retention index (RI) [5], a semi-quantitative parameter that can be derived relatively easy. In general, it is obtained by normalizing late activity concentrations to the integral of the blood time-activity curve. However, RI can be sensitive to cardiac motion, partial volume effects and contribution of intravascular activity and spill-over and therefore may yield inaccurate results. More quantitative results can be obtained by using compartment models, in which correction factors for blood volume and spill-over of activity from the blood can be applied. Interestingly, RI has never been validated using a direct comparison with quantitative results obtained from a full tracer kinetic analysis. Furthermore, blood time-activity curves both with [3,22] and without [23-25] corrections for radioactive metabolites of [^11^C]HED have been used for RI, making it difficult to compare studies. On the other hand, another semi-quantitative measure, standardized uptake value (SUV), could provide a further simplification relative to RI, as it does not require measurement of blood time-activity curves, nor dynamic scanning.

Only few studies [26,27] have performed quantitative analysis of [^11^C]HED data using a single-tissue compartment model, but no systematic assessment of the best tracer kinetic model was performed. Therefore, the aims of this study were to determine the optimal tracer kinetic model for analysing [^11^C]HED data and to assess the validity of several simplifications, such as RI, SUV and a population-averaged metabolite correction.

## Methods

### Patient population

Thirty patients (mean age 67 years, range 43 to 80; 26 males) with ischemic cardiomyopathy and a left ventricular ejection fraction below 35%, as determined by cardiac magnetic resonance imaging (MRI), were included. Ischemic cardiomyopathy was defined as having one or more stenoses >50% on coronary angiography and delayed contrast enhancement on cardiac MRI. The study was approved by the Medical Ethics Review Committee of the VU University Medical Center, and all participants gave written informed consent prior to inclusion.

### Synthesis of [^11^C]HED

Radiolabelled [^11^C]HED was synthesized according to a GMP-compliant procedure (licence nr. 108879 F), using a modification of the method of Van Dort and Tluczek [28]. The starting material, metaraminol (3.0 μmol, 0.5 mg, purchased from ABX, Radeberg, Germany), was dissolved in 100 μL of acetonitrile. [^11^C] Methyl triflate was added to this solution at room temperature, and after reacting for 1 min, this mixture was diluted with 1 mL of 0.1 M ammonium formate in water. The resulting mixture was injected onto a Luna C18 5 μm 250*10 mm high-performance liquid chromatography (HPLC) column (Phenomenex, Torrance, CA, USA), which was eluted with a 95/5 0.1 M ammonium formate/acetonitrile mixture. The product, [^11^C]HED, was eluted at 10 min. This fraction was collected in 60 mL of water for injection. The total solution was passed over a preconditioned (using 5 mL of sterile ethanol (96%) and subsequently 10 mL of water for injection) Oasis WCX cartridge (Waters, Milford, MA, USA). The cartridge was washed with 20 mL of water for injection, and subsequently, the product was eluted from the cartridge with 1.0 mL of sterile ethanol (96%) and 10.0 mL of a sterile and pyrogen-free citrate buffer (3.8 mM citric acid, 5.4 mM sodium citrate, 5.1 mM sodium acetate; pH 5.2). This final mixture was passed over a Millex-GV 0.22 μm filter (Millipore, Billerica, MA, USA), yielding a sterile, isotonic and pyrogen-free solution of 1.6 to 5.1 GBq of [^11^C]HED. The product was analysed using a GraceSmart RP18 5 μm 250*4.6 mm HPLC column (Grace, Columbia, MD, USA), which was eluted with 95/5 0.1 M NaH_2_PO_4_ in a water/acetonitrile mixture. The radiochemical purity was >99% and the specific activity was 54 to 239 GBq · μmol^−1^ at the end of synthesis (*N* = 30).

### Scanning protocol

Studies were performed on a GEMINI TF-64 PET/CT scanner (Philips Healthcare, Best, the Netherlands) [29]. A 60-min emission scan was started simultaneously with the injection of 370 MBq [^11^C]HED, administered as a 5-mL bolus (0.8 mL · s^−1^) followed by a 35-mL saline flush (2 mL · s^−1^). This emission scan was followed immediately by a respiration-averaged low-dose CT (LD-CT) scan (55 mAs, rotation time 1.5 s, pitch 0.825, collimation 16 × 0.625, acquiring 20 cm in 37 s compared to 5 s for a regular LD-CT) during normal breathing.

Images were reconstructed into 36 frames (1 × 10, 8 × 5, 4 × 10, 3 × 20, 5 × 30, 5 × 60, 4 × 150, 4 × 300, 2 × 600 s) using the 3D row action maximum likelihood algorithm and applying all appropriate corrections for scanner normalization, dead time, decay, scatter, randoms and attenuation based on the corresponding LD-CT scan.

### Blood sampling

All patients received an indwelling radial artery catheter for arterial blood sampling during the dynamic emission scan. Using an online detection system [30], arterial blood was withdrawn continuously at a rate of 5 mL · min^−1^ during the first 5 min and 1.7 mL · min^−1^ thereafter. In addition, 7-mL arterial samples were collected manually at 2.5, 5, 10, 15, 20, 30, 40 and 60 min post injection and analysed for plasma and whole blood activity and the presence of radiolabelled metabolites, similarly as described in [31]. For each sample, activity concentrations in plasma and whole blood were determined. Furthermore, plasma was analysed for radiolabelled metabolites of [^11^C]HED by solid-phase extraction (SPE). In brief, 1 mL of plasma was diluted with 2 mL water and loaded onto an activated Oasis WCX cartridge (Waters, Milford, MA, USA, 3 mL, 60 mg). First, the cartridge was washed with 3 mL of 1% acetonitrile in water and subsequently eluted with 3 mL of 1 M HCl/ethanol (95/5). Radioactivity in all three fractions (plasma, 1% acetonitrile in water, 1 M HCl/ethanol) was measured. The first two fractions represent polar radiolabelled metabolites of [^11^C]HED and the third intact [^11^C]HED. No other metabolites were observed in the third fraction, and therefore, further analysis using HPLC was not required.

### Input functions

Blood sampler data were corrected for delay and dispersion by fitting the early part of the sampler curve to the ascending aorta (AA) time-activity curve (TAC) using a single-tissue compartment model with additional parameters for delay and dispersion constants [32]. The AA was chosen as it was shown to provide more reproducible curves than either the left ventricle or the left atrium [33]. This region was obtained by drawing 1-cm-diameter circular regions of interest (ROIs) over the AA in at least five consecutive transaxial images of an early frame of the dynamic scan, i.e. the frame in which the first pass of the bolus through the AA was best visible [34]. To avoid partial volume effects, ROIs were placed in the centre of the AA, with ROI borders at least 5 mm from the edge of the AA. Next, the resulting delay- and dispersion-corrected sampler curve was calibrated using the manually drawn blood samples. Plasma/whole blood ratios derived from the manual blood samples were fitted to a sigmoid function, as described previously [35]. Similarly, parent fractions derived from the same samples were also fitted to a sigmoid function. Finally, the plasma blood sampler input function (BSIF) *C*_P_(*t*) was obtained by multiplying the delay- and dispersion-corrected and calibrated blood TAC with both these sigmoid functions.

In addition to these individual corrections, a population-averaged correction for both plasma/whole blood ratios and parent fractions was obtained using data from ten randomly selected patients, which was applied to all delay- and dispersion-corrected and calibrated blood TAC.

To correct for spill-over from the right ventricle (RV), a set of ROIs was placed over the RV cavity in five consecutive transaxial planes, with ROI boundaries at least 1 cm away from the RV wall to avoid spill-over of myocardial activity. These ROIs were combined in an RV VOI, which was then transferred to the full dynamic image sequence to obtain the right ventricular time-activity curve.

### Data analysis

Using software developed in-house within MATLAB 7.0.4 (MathWorks, Natick, MA, USA), 16 myocardial segment VOIs were drawn on the final frame of the dynamic scan according to the 17-segment model of the American Heart Association, excluding the apex [36]. This VOI template was projected onto the entire dynamic emission scan to extract segment TACs. Segment TACs were fitted to a single-tissue compartment model (1T2k) and both an irreversible (2T3k) and reversible (2T4k) two-tissue compartment model using non-linear least squares regression. No additional compartments for metabolites within the heart were used, as presence of metabolites in the heart was found to be negligible in a previous preclinical study [4]. Corrections for spill-over from left and right ventricular cavities were included in all models as follows, based on the findings in the Appendix:

1 $$ {C}_{\mathrm{PET}}(t)={C}_{\mathrm{T}}(t)+{V}_{\mathrm{A}}\cdot {C}_{\mathrm{A}}(t)+{V}_{\mathrm{RV}}\cdot {C}_{\mathrm{RV}}(t) $$

in which *C*_PET_(*t*) represents the measured concentration, *C*_T_(*t*) the myocardial tissue concentration, *C*_A_(*t*) the arterial whole blood concentration and *C*_RV_(*t*) the right ventricular cavity concentration. In Equation 1, RV activity is treated as spill-over and the *V*_A_ contribution is assumed to be due to spill-over from the left ventricular cavity, rather than due to arterial activity within the myocardium. Clearly, it is not possible to distinguish between contributions from true myocardial blood volume and spill-over from the left ventricle, but previously, it has been shown that assuming *V*_A_ to be due to spill-over rather than to myocardial arterial blood volume provided more stable results for [^15^O] water [37], which was confirmed by simulation studies for [^11^C]HED (Appendix).

Differences in variances of measured activity in each frame were accounted for by including the following frame weighing factors *W* during the fitting process:

2 $$ W=\frac{L^2}{C\cdot {f}^2} $$

in which *L* is the length of each frame, *C* the total number of counts of each frame and *f* the decay correction factor of each frame. Weights were normalized such that the average of all weighing factors was equal to unity.

Outcome parameters for each model are listed in Table 1 and were defined as in [38]. With the exception of *V*_S_, all parameters were used as independent variables during the fitting process.

**Table 1:** **Outcome parameters for various models**

	**All models**	**1T2k model**	**2T3k model**	**2T4k model**		
**Outcome parameter**	**Unidirectional influx rate**	**Total volume of distribution**	**Total rate of irreversible binding**	**Total volume of distribution**	**Non-displaceable binding potential**	**Specific volume of distribution**
Symbol	*K*_1_	*V*_T,1t_	*K*_*i*_	*V*_T,2t_	BP_ND_	*V*_S_
Definition	-	$\frac{K_1}{k_2}$	${K}_1\cdot \frac{k_3}{k_2+{k}_3}$	$\frac{K_1}{k_2}\cdot \left(1+\frac{k_3}{k_4}\right)$	$\frac{k_3}{k_4}$	$\frac{K_1}{k_2}\cdot \frac{k_3}{k_4}$

To exclude outliers and unreliable fits, standard deviations of outcome parameters were estimated. Only outcome parameters with a coefficient of variation (CoV; standard deviation divided by the final outcome parameter) <25% were used for further analysis. For each model, the number of fits with stable parameters was used as an outcome measure.

For each segment, the best fit was determined using the Akaike information criterion (AIC [39]), the Schwarz criterion (SC [40]) and the *F*-test. AIC, SC and the *F*-statistic were defined according to Equations 3, 4 and 5, respectively:

3 $$ \mathrm{A}\mathrm{I}\mathrm{C}=N\cdot \ln \left(\mathrm{WSSE}\right)+2\cdot p $$

4 $$ \mathrm{S}\mathrm{C}=N\cdot \ln \left(\mathrm{WSSE}\right)+p\cdot \ln (N) $$

5 $$ F=\frac{\left\{\frac{\left({\mathrm{WSSE}}_1-{\mathrm{WSSE}}_2\right)}{\left({p}_2-{p}_1\right)}\right\}}{\left\{\frac{{\mathrm{WSSE}}_2}{\left(N-{p}_2\right)}\right\}} $$

in which *N* is the number of frames (36 in the present study), WSSE the weighted squared sum of errors, using the weights of Equation 2, and *p* the total number of parameters for each model, i.e. 4, 5 and 6 for 1T2k, 2T3k and 2T4k, respectively, including spill-over fractions. The subscripts 1 and 2 in Equation 5 represent the model with the highest (2) and the lowest (1) number of parameters. For the *F*-test, an *F*-statistic with a *p* value <0.05 was considered to correspond to a significant improvement in the goodness of fit.

Correlations between volumes of distribution (*V*_T_) of various models and between individual and population-averaged metabolite corrections were assessed using linear regressions. Agreement between different *V*_T_ and metabolite corrections were assessed using intraclass correlation coefficients (ICC). For validation of the population-averaged metabolite correction, patients who were used to define this population average were not included in the comparison between individual and population corrections.

Finally, RI and SUV were calculated using the last 10 min of the tissue TAC normalized to the integral of the parent plasma TAC (RI_P_) or of the uncorrected whole blood TAC (RI_WB_) and to the injected dose divided by patient weight (SUV), respectively.

### Simulations

Simulations were performed using input functions of a randomly selected patient. Tissue TACs (*C*_PET_(*t*)) were calculated using the reversible two-tissue model for healthy, scar and viable but denervated tissues. Based on clinical data obtained after a pilot analysis of five patients, values for *K*_1_, *k*_2_, *k*_3_ and *k*_4_ were set at 0.6 mL · cm^−3^ · min^−1^, 0.15 min^−1^, 0.3 min^−1^ and 0.02 min^−1^ for healthy tissue, at 0.2 mL · cm^−3^ · min^−1^, 0.1 min^−1^, 0.15 min^−1^ and 0.05 min^−1^ for scar tissue, and at 0.6 mL · cm^−3^ · min^−1^, 0.15 min^−1^, 0.15 min^−1^ and 0.05 min^−1^ for viable but denervated tissue, respectively. An arterial spill-over fraction of 0.3 and a right ventricular spill-over fraction of 0.1 (dimensionless) were assumed in all settings. Different levels of Gaussian noise (1%, 5%, 10% and 20%, representing noise on the whole LV level, the coronary level, the segmental level and the voxel level, respectively) were added to tissue TACs, after which they were fitted using non-linear least squares to each of the three compartment models. To correct for differences in variance of activity in each frame, noise was weighted according to Equation 2 and the average noise of the final six frames was set to the aforementioned values of noise levels. For each noise level, this process was repeated 1,000 times. Finally, average values of the various outcome parameters together with their CoV were obtained. Bias in derived parameters was obtained by comparing all obtained parameters with those estimated using the reversible two-tissue model.

## Results

### Compartment models

#### Clinical data

**Figure 1:**
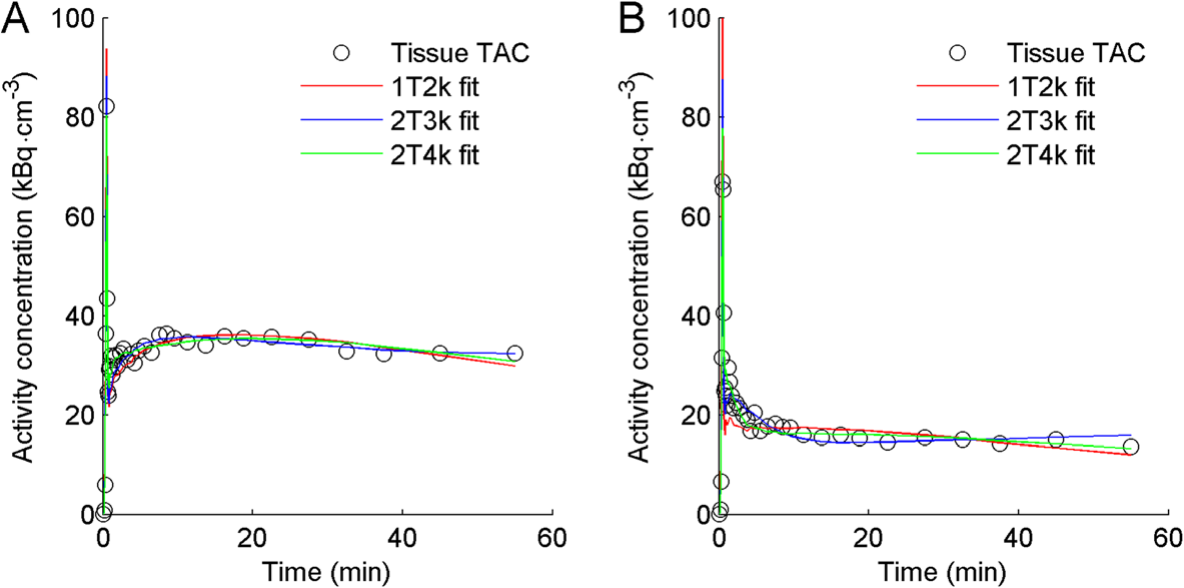
**Typical time-activity curves of heart segments with (A) high and (B) low uptake in the same patient.** Red, blue and green lines represent best fits according to reversible single-tissue (1T2k), irreversible two-tissue (2T3k) and reversible two-tissue (2T4k) models, respectively.

For one patient, online sampling could not be performed due to technical problems, and this patient was excluded from the study. Typical examples of myocardial TACs with high and low uptake, together with corresponding fits, are shown in Figure 1. Based on AIC, the 2T4k, 2T3k and 1T2k models were preferred in 364 (75.8%), 79 (16.4%) and 37 (7.7%) segments, respectively. Based on SC, this was 324 (67.5%), 99 (20.6%) and 57 (11.9%), respectively. Finally, the *F*-test showed a significantly improved fit when using the 2T4k model instead of both 2T3k and 1T2k models in 88.5% and 100% of all segments, respectively, indicating that the 2T4k model is the most appropriate model. Both 1T2k and 2T3k show systematic errors in the obtained fits (runs test *p* value <0.001 for both). No systematic error in 2T4k fits was observed (*p* = 0.06).

In 76.9%, 96.9% and 99.6% of all segments, *K*_1_ was fitted with a CoV <25% for 2T4k, 2T3k and 1T2k models, respectively. For 2T4k-derived *V*_T_ (*V*_T,2t_), *V*_S_ and BP_ND_, 2T3k-derived *K*_*i*_ and 1T2k-derived *V*_T_ (*V*_T,1t_), a CoV <25% was obtained in 77.1%, 77.1%, 23.3%, 86.0% and 94.6% of all segments, respectively. Due to the very low number of BP_ND_ values with a sufficiently low CoV, BP_ND_ was excluded from further analysis.

#### Simulations

Simulations showed that, based on both AIC and SC, preference for the simpler single-tissue model increased with increasing noise levels (Table 2). Tables 3 and 4 provide accuracy and precision of *V*_T,1t_, *K*_*i*_, *V*_T,2t_ and *K*_1_ estimates for increasing noise levels. In general, accuracy and precision of *V*_T,2t_ and *K*_1_ derived using 2T4k decreased rapidly with increasing noise levels. At increasing noise levels, the best accuracy and precision were obtained for *V*_T,1t_ followed by *K*_*i*_, especially for scar and viable but denervated tissue. The 1T2k model also provided the highest precision of *K*_1_, especially for tissues with low uptake, and the lowest noise-induced bias.

**Table 2:** **Model preference (%) at various noise levels according to AIC, SC and** ***F*** **-test**

	**AIC**			**SC**			***F*** **-test**		
**Noise (%)**	**1T2k**	**2T3k**	**2T4k**	**1T2k**	**2T3k**	**2T4k**	**2T4k over 2T3k**	**2T4k over 1T2k**	**2T3k over 1T2k**
1	0.00	0.00	100.00	0.00	0.00	100.00	100.0	100.00	100.00
5	0.00	5.57	94.43	0.03	8.73	91.23	93.07	99.63	93.17
10	4.73	27.70	67.57	11.30	33.77	54.93	55.47	80.43	79.70
20	22.60	47.00	30.40	35.17	47.87	16.97	23.40	36.13	46.93

Numbers represent the percentage of simulations for which the specific model yielded the lowest AIC or SC or, for the *F*-test, the percentage of simulations for which the specific model yielded a significantly (*p* < 0.05) better fit than the simpler model.

**Table 3:** **Average bias (%) for all parameters derived from simulated tissue TACs of all three simulated tissues**

	**Noise**	***K***_**1**_ **1T2k**	***V***_**T**_ **1T2k**	***K***_**1**_ **2T3k**	***K***_***i***_ **2T3k**	***K***_**1**_ **2T4k**	***V***_**T**_ **2T4k**
Healthy	0%	−26.37	−22.01	−19.00	21.51	0.00	0.00
	1%	−26.54	−21.58	−18.99	21.52	0.01	0.31
	5%	−26.47	−20.97	−18.43	22.36	0.47	7.41
	10%	−26.37	−18.03	−15.01	27.49	3.76	14.49
	20%	−26.17	−10.74	−6.73	39.91	8.23	15.56
Scar	0%	−22.07	−11.97	−15.27	41.21	0.00	0.00
	1%	−22.26	−11.88	−15.26	41.23	0.00	0.20
	5%	−22.26	−11.78	−15.21	41.32	0.57	14.19
	10%	−22.32	−10.73	−14.50	42.50	3.12	95.17
	20%	−21.79	−8.99	−11.65	47.25	14.08	208.98
Denervated	0%	−29.56	−12.35	−20.71	58.59	0.00	0.00
	1%	−29.79	−12.35	−20.68	58.63	0.03	0.02
	5%	−29.74	−12.19	−20.72	58.56	0.28	1.24
	10%	−29.68	−12.13	−20.39	59.22	0.38	18.55
	20%	−29.52	−10.95	−18.95	62.10	4.33	82.07

Bias was calculated by comparing the obtained values of *K*_1_, *V*_T_ and *K*_*i*_ with those used in the 2T4k model to generate time-activity curves (i.e. the 2T4k model has no bias for 0% noise). Bias due to noise can be appreciated by comparing the obtained bias with those at 0% noise.

**Table 4:** **CoV (%) for all parameters derived from simulated tissue TACs of all three simulated tissue classes**

	**Noise**	***K***_**1**_ **1T2k**	***V***_**T**_ **1T2k**	***K***_**1**_ **2T3k**	***K***_***i***_ **2T3k**	***K***_**1**_ **2T4k**	***V***_**T**_ **2T4k**
Healthy	1%	0.41	1.97	0.98	1.85	1.39	3.96
	5%	1.98	10.41	5.06	17.22	6.95	21.32
	10%	3.94	23.02	10.71	36.97	11.61	28.82
	20%	7.64	38.87	21.74	48.47	17.21	35.20
Scar	1%	0.41	0.93	0.63	4.28	1.09	1.71
	5%	2.08	4.29	3.17	25.10	5.47	65.24
	10%	4.24	9.22	5.89	48.29	9.77	136.43
	20%	8.64	21.34	10.46	68.38	23.92	133.26
Denervated	1%	0.36	0.65	0.52	2.93	0.76	1.08
	5%	1.74	3.02	2.69	15.53	3.91	8.52
	10%	3.60	6.32	4.92	32.84	7.88	68.26
	20%	6.82	13.45	9.75	56.64	16.11	99.17

#### Single-tissue vs two-tissue compartment model

**Figure 2:**
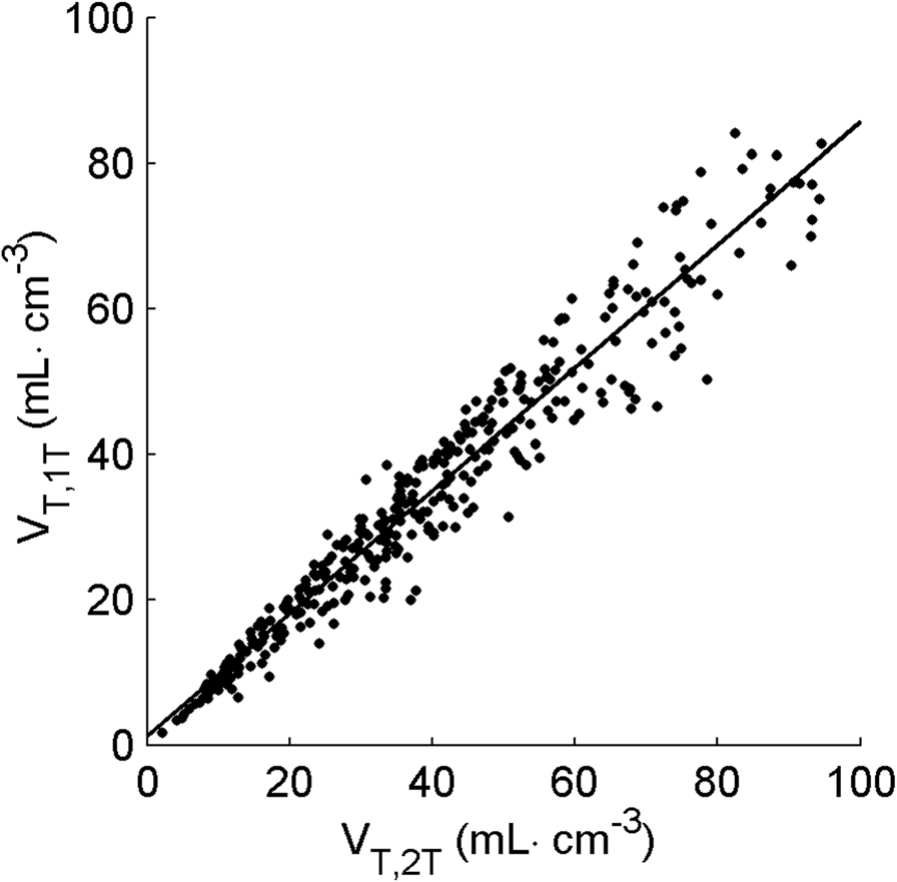
**Correlation of** ***V***_**T,1t**_ **(vertical axis) with** ***V***_**T,2t**_ **(horizontal axis) for clinical data.**

The correlation between *V*_T,1t_ and *V*_T,2t_ is shown in Figure 2 for clinical data. Both correlation (*r*^2^ = 0.94, slope = 0.84) and agreement (ICC = 0.96) between *V*_T,1t_ and *V*_T,2t_ were high. *V*_T,1t_ was significantly lower than *V*_T,2t_, as indicated by the slope, which was significantly different from 1 (*p* < 0.001). These results indicate that *V*_T_ obtained with the 1T2k model yields the same information as that obtained with the 2T4k model, albeit with a consistent underestimation. Consequently, based on its better precision, *V*_T,1t_ was used to validate simplifications.

### Simplifications

**Figure 3:**
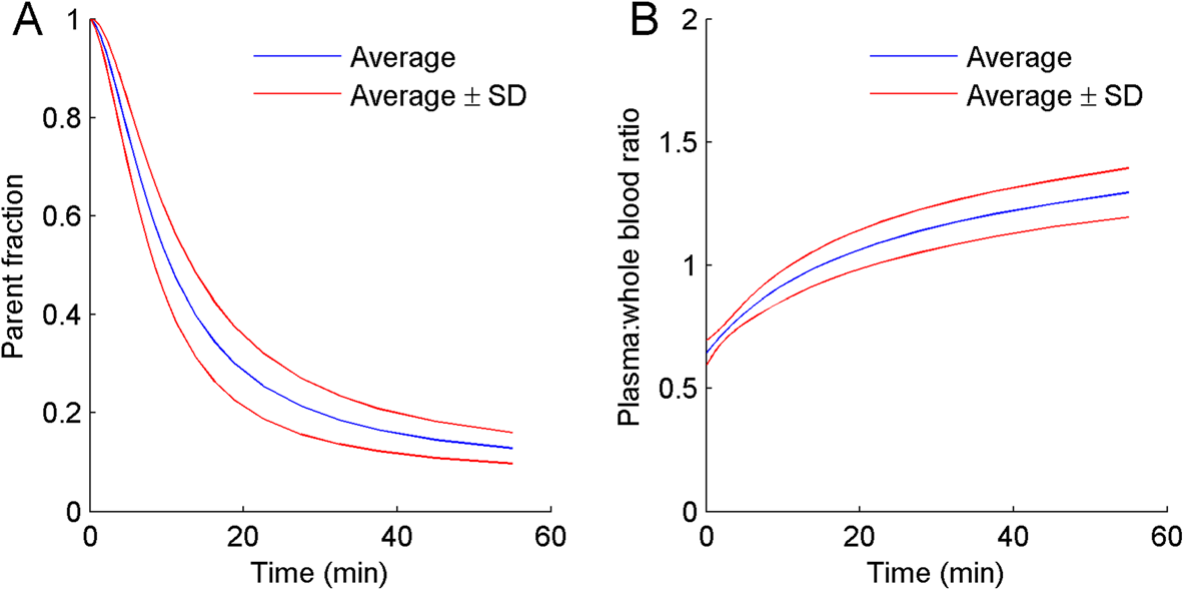
**Parent fraction in arterial plasma (A) and plasma/whole blood concentration ratio (B) as a function of time.** Blue lines indicate mean values and red lines mean values ± one standard deviation.

**Figure 4:**
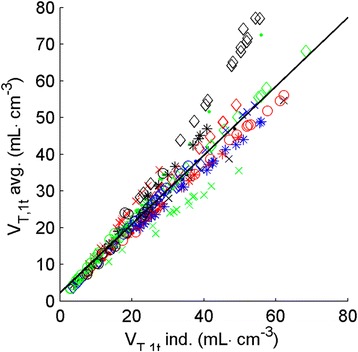
**Correlation plot of** ***V***_**T,1t**_ **calculated using individual metabolite corrections (horizontal axis) and population-averaged metabolite correction (vertical axis).** Each patient (*n* = 20) is represented by its own symbol.

Figure 3 shows mean parent [^11^C]HED fractions and plasma/whole blood ratios as a function of time. The corresponding standard deviations indicate substantial variation between patients. Figure 4 shows a scatter plot of *V*_T,1t_ calculated with individual and population-averaged metabolite corrections. Correlation (*r*^2^ = 0.89) and agreement (ICC = 0.94) were high and the slope of the linear fit was 0.98. However, there was significant bias in several patients when the population-averaged metabolite correction was used, ranging from −28% to 34% for individual patients.

**Figure 5:**
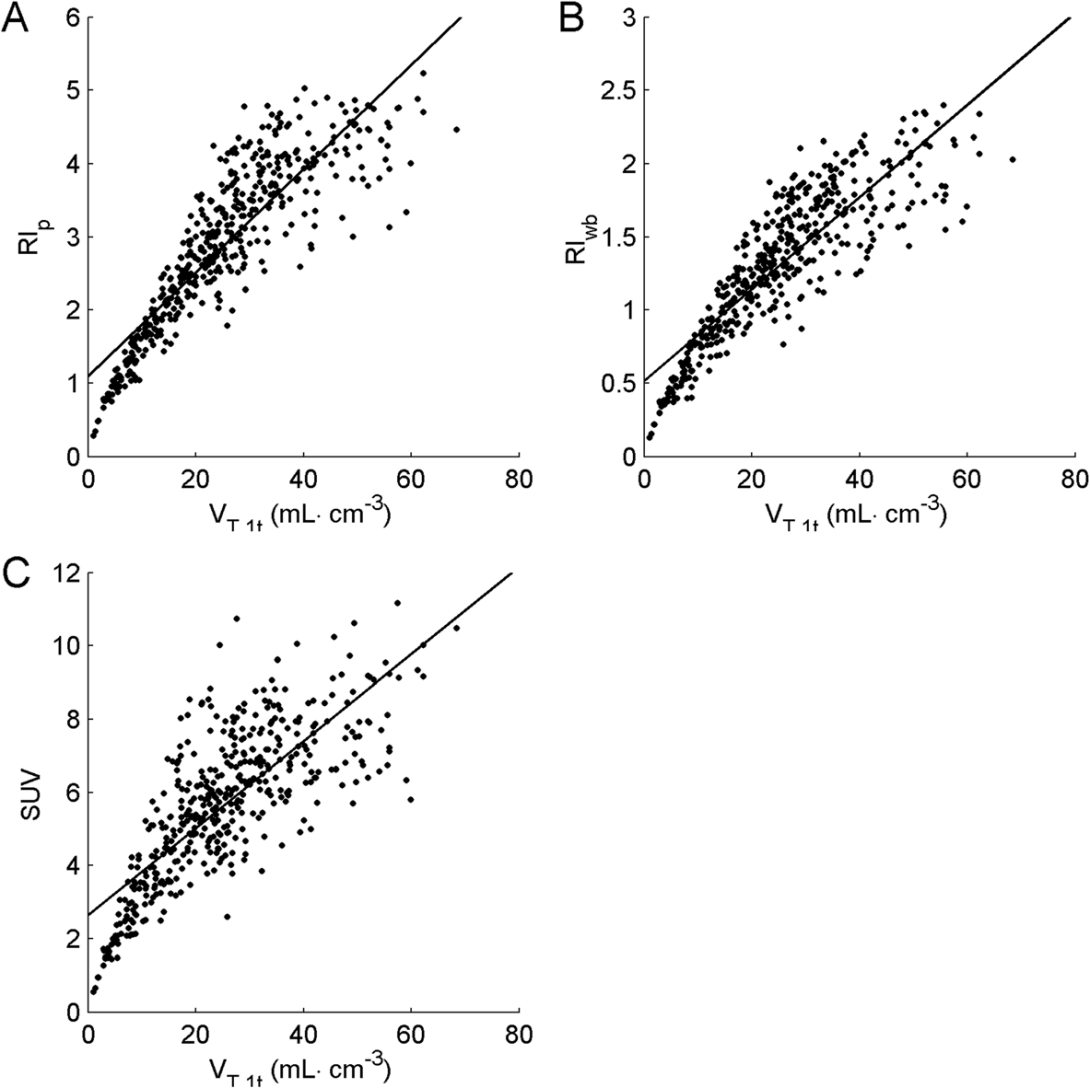
**RI**_**P**_ **(A), RI**_**WB**_ **(B) and SUV (C) as a function of corresponding** ***V***_**T,1t**_ **.** RI_P_, RI_WB_ and SUV were obtained by normalizing radioactivity concentrations between 50 and 60 min post injection to the total amount of [^11^C]HED in plasma (RI_P_), total amount of radioactivity in whole blood (RI_WB_) and injected dose per patient weight (SUV), respectively.

Figure 5 shows relationships of RI_P_, RI_WB_ and SUV with *V*_T,1t_. There was a clear and strong correlation of RI_P_ and RI_WB_ with *V*_T,1t_ (*r*^2^ = 0.77 for RI_P_ and *r*^2^ = 0.76 for RI_wb_). Correlation of SUV with *V*_T,1t_ was significantly lower (*r*^2^ = 0.62). It is clear, however, that all three relationships were non-linear.

## Discussion

In the present study, the optimal tracer kinetic model for kinetic analysis of [^11^C]HED scans was identified and several commonly used simplifications were evaluated. First, Akaike and Schwarz criteria clearly indicated that, for both clinical and simulated (Table 2) data, the reversible two-tissue model was the preferred model for describing [^11^C]HED kinetics. Similar results were obtained when using the *F*-test, which is insensitive to any potential bias introduced in AIC and SC due to the scaling factors as presented in Equations 3 and 4. This confirms that, based on goodness of fit, the reversible two-tissue model was the optimal model. Simulations, on the other hand, also indicated that with increasing noise levels (starting at 5% noise, corresponding with noise at the level of the coronary territory), precision of all parameter estimates using this model rapidly decreased (Table 3) and that, for higher noise levels, model preference shifted towards models with fewer parameters (Table 2). In addition, for clinical data, the number of reliable estimates was lower for the 2T4k parameters *V*_T,2t_, *V*_S_ and BP_ND_ (77.1, 77.1 and 23.3%, respectively) than for *K*_*i*_ derived using the 2T3k model (86.0%) and *V*_T,1t_ obtained using the 1T2k model (94.6%). The large number of estimates with high uncertainty indicates that the use of the reversible two-tissue model may not be feasible in routine clinical practice, as it would mean discarding over 20% of all data. In addition, most estimates with high uncertainty in 2T4k parameters were in regions with reduced [^11^C]HED uptake, indicating that the 2T4k model is especially vulnerable in diseased myocardium, which significantly limits the applicability of this model.

For the reversible two-tissue model, the total volume of distribution can be separated in specific and non-specific volumes of distribution. In this study, the total volume of distribution approximated the specific volume of distribution and enabled the use of the simpler single-tissue model, as the contribution of the non-specific compartment was very small, if not negligible. As shown in Figure 2, *V*_T,1t_ yields similar results to *V*_T,2t_, indicating that the single-tissue model yields similar information to the reversible two-tissue model. In addition, precision was much higher for *V*_T,1t_ than for *V*_T,2t_ (Tables 3 and 4) and noise-induced biases were lower for *V*_T,1t_. Since the single-tissue model is simpler, more stable and less sensitive to noise compared with the reversible two-tissue model, it is preferred for further use, especially for smaller regions or regions with relatively low uptake.

**Figure 6:**
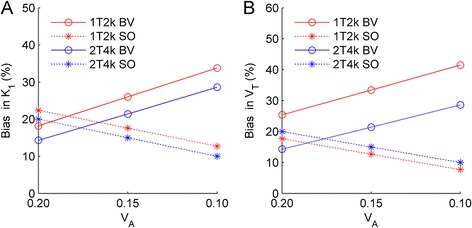
**Bias due to interpretation of blood activity.** Bias in % for estimates of *K*_1_ **(A)** and *V*_T_ **(B)** derived using 1T2k (red) and 2T4k (blue) models using interpretations *BV* (continuous lines) and *SO* (dashed lines) for *healthy tissue*. Please note that the sum of *V*_A_ and *V*_SO_ was set to 0.3 for all simulated tissue curves.

In this study, arterial activity was assumed to be spill-over from the arterial blood pool, rather than activity originating from arterial blood within the tissue. Both assumptions are physiologically not fully correct and ideally, a combination should be used. However, differentiating between contributions from spill-over and myocardial blood volume is impossible, as kinetics of both contributions are identical. Previously, it has been shown that, in a comparison of MBF as measured with [^15^O] water against labelled microspheres, assuming arterial activity to be spill-over yields more accurate results [37], at least for [^15^O] water. A simulation study (see Figure 6 in Appendix) showed that the same is true for HED, although a bias was found in both implementations. Bias was smallest when arterial activity was assumed to be due to spill-over. In addition, arterial blood volume fraction within myocardial tissue is relatively constant in the myocardium [41], and therefore, it is expected that bias in the spill-over implementation, which is dependent on the magnitude of this blood volume fraction, is more consistent than bias in the tissue blood volume implementation, which depends on the recovery of the scanner and segment size.

The use of HED as an innervation tracer has recently been debated [42], where it was suggested that HED is a flow-limited rather than innervation-limited tracer. This was suggested to lead to the impossibility to fit HED data using standard kinetic modelling techniques and make HED uptake insensitive to early changes in innervation. However, when performing tracer kinetic analyses, parameters representing innervation (i.e. *K*_*i*_, *V*_T_, *V*_S_ and BP_ND_) can be obtained together with parameters representing flow and extraction (i.e. *K*_1_). This enables separating flow effects from innervation effects by studying both *K*_1_ and, when the 1T2k model is used, *V*_T_. Furthermore, in this study we show that, utilizing plasma input functions fully corrected for radiolabelled metabolites and plasma-to-blood concentration ratios, HED could reliably be fitted using a 2T4k model or, with slightly poorer fits, using a 1T2k model, which is in contrast to the suggestion in [42].

The non-linear relation between RI and *V*_T_, however, does indicate that for RI it might be the case that early changes are not readily detected. Indeed, when a sensitivity analysis is performed for changes in *k*_3_, which represents HED uptake through the norepinephrine transporter, using *K*_1_, *k*_2_ and *k*_4_ of 0.6 mL · cm^−3^ · min^−1^, 0.15 min^−1^ and 0.02 min^−1^, respectively, and gradually decreasing *k*_3_, RI showed a lower and non-linear sensitivity to changes in innervation (see Additional file 1). On the other hand, *V*_T,1t_ showed good sensitivity to changes in innervation, where decreases in *V*_T,1t_ were similar to decreases in *k*_3_, confirming that *V*_T,1t_ is a more reliable measure of innervation than RI. Nevertheless, sensitivity of RI to changes in *k*_3_ may still be sufficient for routine clinical use as large reductions in *k*_3_ are readily detected by RI. It is also important to note that another recent study [43] showed that HED was able to detect early changes in innervation, confirming that HED is likely to represent innervation even when using RI.

Potentially, a population-averaged metabolite correction could, in combination with an image-derived input function, make arterial cannulation obsolete. There was, however, substantial variation in individual metabolism (Figure 3), resulting in large errors of up to 30% in fitted parameters when using a population-averaged metabolite correction (Figure 4) for approximately 20% of the patients. Clearly, for absolute quantification, a population-averaged metabolite correction is not feasible, at least not in the case of [^11^C]HED, as a method that produces inaccurate results in as much as 20% of the cases should be discarded. However, when relative differences within a single patient are of interest, e.g. when assessing defect areas (percentage of LV with *V*_T_ below a reference value), population-averaged metabolite corrections can be considered. Using venous samples for metabolite correction might be an alternative for both relative analysis and absolute quantification, but this requires further validation.

In addition, the use of an image-derived input function would be preferred over the use of online blood sampling. However, image-derived input functions require validation for each individual radiotracer and preliminary results for [^11^C]HED [44] have shown that late activity concentrations were overestimated in the image-derived input functions, which is in line with observations in other studies that made use of a combination of image-derived input function and blood sampler data [26,27]. Given the fact that an image-derived input function can be used for other tracers such as [^15^O] water [45] and [^18^ F] FDG [33], the particular biodistribution of HED with its high contrast between the myocardium and the blood at later time points, together with high liver uptake, will require improved reconstruction methods and/or scatter correction algorithms before an image-derived input function can be used for [^11^C]HED.

A reasonable correlation was found between *V*_T,1t_ and either RI_P_, RI_WB_ or SUV (*r*^2^ = 0.77, 0.76 and 0.62, respectively). These correlations suggest that RI_P_, RI_WB_ and SUV may be used to distinguish innervated from denervated regions of the myocardium. However, it should be noted that relationships were non-linear (Figure 5). Consequently, these simplified measures will be less sensitive in detecting early changes in regions with high innervation, e.g. in early disease, or for monitoring response during treatment. In these cases, the use of quantitative measures is recommended. On the other hand, for routine clinical (diagnostic) use, RI might be sufficient, as large reductions in HED uptake are reflected in large reductions in RI. In addition, simplified methods may lead to biased estimates of defect sizes, as these usually are derived using cut-off values, which are based on a percentage of uptake in a reference region within the heart. Due to the non-linear relationships, cut-off values for RI_P_, RI_WB_ and SUV will be different from those for *V*_T,1t_ and, consequently, defect areas may differ significantly.

In the present study, RI was based on uptake between 50 and 60 min post injection, whilst 30 to 40 min is more commonly used. When RI was calculated using uptake from 30 to 40 min post injection, similar results were obtained (*r*^2^ = 0.72 and *r*^2^ = 0.73 for RI_P_ and RI_WB_, respectively). For RI_P_, this correlation was slightly, but significantly, lower than that for the 50 to 60 min uptake period (*p* = 0.043). For RI_WB_, this difference was not statistically significant (*p* = 0.071). Although correlation with *V*_T,1t_ was still reasonable, based on these findings, it is recommended to use later times for measuring RI due to the slow kinetics of HED.

Finally, it is important to note that the aforementioned results and interpretations are made from a modelling point of view, i.e. which parameter describes the measured HED activity best. This might be different from which parameter actually describes myocardial presynaptic activity best. Ideally, a blocking study or animal study should be performed to compare the obtained parameters with a gold standard measurement. In addition, the performance of the quantitative parameters used in the present study needs to be assessed in clinical studies, as the added clinical value of *V*_T_ over simplified measures such as RI_P_ still needs to be defined. Whilst simulations (see Additional file 1) show that quantitative measures are more sensitive to changes in *k*_3_, the rate constant reflecting norepinephrine transport, RI also showed significant correlation with *k*_3_, suggesting that RI may be sufficiently sensitive for measuring (changes in) innervation. Further clinical validation studies using RI_P_, RI_WB_, SUV and *V*_T_ are warranted.

## Conclusions

Although a reversible two-tissue compartment model best describes [^11^C]HED kinetics, a single-tissue compartment model is preferred for routine clinical studies, as it is more robust at clinically relevant noise levels and, at the same time, provides *V*_T_ results that are highly correlated with those obtained with the two-tissue compartment model. Simplified measures, such as RI and SUV, showed good correlation with fully quantitative results and may be used to detect regions of denervation, although the non-linear relationship with *V*_T_ might limit their application in, for instance, monitoring response to treatment.

## Appendix

Contribution of arterial activity measured within a myocardial region is typically the combination of two different effects: a physical blood volume fraction within the myocardium and spill-over of activity from the left ventricular cavity into the myocardium. This can be represented as follows:

A1 $$ {C}_{\mathrm{PET}}(t)=\left(1-{V}_{\mathrm{A}}\right)\cdot {C}_{\mathrm{T}}(t)+\left({V}_{\mathrm{A}}+{V}_{\mathrm{LV}}\right)\cdot {C}_{\mathrm{A}}(t)+{V}_{\mathrm{RV}}\cdot {C}_{\mathrm{RV}}(t) $$

in which *C*_T_(*t*) represents the HED concentrations in the myocardial tissue itself whilst *C*_PET_(*t*), *C*_A_(*t*) and *C*_RV_(*t*) represent the measured regional, arterial and right ventricular blood activity concentrations, respectively. *V*_A_ represents the actual arterial blood volume fraction in the tissue, and *V*_LV_ and *V*_RV_ represent the spill-over fractions from the left and the right ventricular cavity, respectively. During compartment modelling, it is not possible to distinguish *V*_A_ from *V*_LV_, and therefore, the estimated arterial activity measured within a myocardial region can be interpreted in two different ways:

A2 $$ {C}_{\mathrm{PET}}(t)={C}_{\mathrm{T}}(t)+{V}_{\mathrm{LV}}\cdot {C}_{\mathrm{A}}(t)+{V}_{\mathrm{RV}}\cdot {C}_{\mathrm{RV}}(t) $$

A3 $$ {C}_{\mathrm{PET}}(t)=\left(1-{V}_{\mathrm{A}}\right)\cdot {C}_{\mathrm{T}}(t)+{V}_{\mathrm{A}}\cdot {C}_{\mathrm{A}}(t)+{V}_{\mathrm{RV}}\cdot {C}_{\mathrm{RV}}(t) $$

Interpretation *SO* (spill-over, Equation A2) assumes all measured arterial activity in the tissue regions to be due to spill-over from the left ventricular cavity, and a correction factor *V*_A_ · *C*_A_(*t*) is used to correct for this spill-over fraction. In contrast, interpretation *BV* (blood volume, Equation A3) assumes all arterial activity in the tissue regions to be due to intravascular activity originating from the arterial blood volume within the myocardium itself. Since measured *K*_1_ represents *K*_1_ in the myocardial tissue only, a correction factor for tissue fraction has to be applied in model *BV*, in addition to the correction factor *V*_A_ · *C*_A_(*t*). This tissue fraction is estimated as 1 − *V*_A_. In reality, measured activity originates from both spill-over from the left ventricular cavity and myocardial arterial blood, but it is impossible to distinguish between both contributions and, in practice, only a single correction can be applied. It is important to note that the actual fits obtained using Equations A2 and A3 are identical and only the interpretation of *K*_1_ and consequently *V*_T_ is different.

To define the optimal model implementation, a simulation study was performed for both model interpretations. Three tissue curves (i.e. *healthy tissue*, *scar tissue* and *viable but denervated tissue*) were defined, using the same parameters as for the noise simulations. *V*_RV_ was 0.1 for all simulated tissue curves. For each of the three tissue curves, arterial activity was simulated to be due to both spill-over from the left ventricular cavity (*V*_LV_) and arterial blood volume in the myocardium (*V*_A_) using Equation A1.

The total contribution of arterial blood activity was set to 0.3, based on clinical data (average *V*_A_ for all segments 0.30 ± 0.10, 0.28 ± 0.10 and 0.25 ± 0.11 for 1T2k, 2T3k and 2T4k models, respectively), and *V*_RV_ was set to 0.1, again based on clinical data (average *V*_RV_ for all segments 0.07 ± 0.08, 0.06 ± 0.07 and 0.06 ± 0.07 for 1T2k, 2T3k and 2T4k models, respectively, and for septal segments alone 0.14 ± 0.09, 0.13 ± 0.09 and 0.13 ± 0.09 for 1T2k, 2T3k and 2T4k models, respectively). For each tissue curve, a simulation was performed with *V*_A_ set to 0.1, 0.15 and 0.2 and *V*_LV_ to 0.2, 0.15 and 0.1, respectively. In addition, for each tissue curve, *V*_A_, *V*_LV_ and *V*_RV_ were set to 0 and a fit was performed to obtain estimates without any confounding effects of *V*_A_, *V*_LV_ and *V*_RV_, and these values were used as reference values. Then, the obtained time-activity curves were fitted using both Equations A2 and A3 and the reversible one- and two-tissue compartment models, and the obtained *K*_1_ and *V*_T_ were compared with the reference values. For healthy tissue, bias in the obtained results is shown in Figure 6. Similar results were obtained for scar and viable but denervated tissue. As expected, bias was observed for both *BV* and *SO*. In general, magnitude and variation in bias were larger for *BV* than for *SO*, indicating that estimates obtained with *SO* are more stable and closer to the true simulated tissue values. For these reasons, *SO* was considered to be the most robust model interpretation. In addition, *V*_A_ is relatively constant in the myocardium (around 10% [41]), and therefore, it is likely that spill-over has a larger and more variable contribution to *V*_A_. Therefore, it is expected that bias in *SO*, which is dependent on the magnitude of *V*_A_, is also relatively constant. In contrast, bias in *BV* is dependent on the magnitude of *V*_LV_, which depends on scanner resolution, segment size and thickness of the myocardial wall, and therefore, it is expected to vary between patients and scans. For these reasons, *SO* was used in the present study.

## Additional file

Additional file 1: Figure S1. Results of sensitivity analysis, showing relative change in *k*_3_ (*x*-axis, representing transport through the norepinephrine transporter) and resulting relative changes in fitted outcome measures. Dashed line represents line of identity.
