# Supplementary figures and images for: Quantification of [11C]-meta-hydroxyephedrine uptake in human myocardium
Source: EJNMMI Res. 2014 Sep 26;4:52. doi: 10.1186/s13550-014-0052-4 (PMC4452641; doi:10.1186/s13550-014-0052-4)

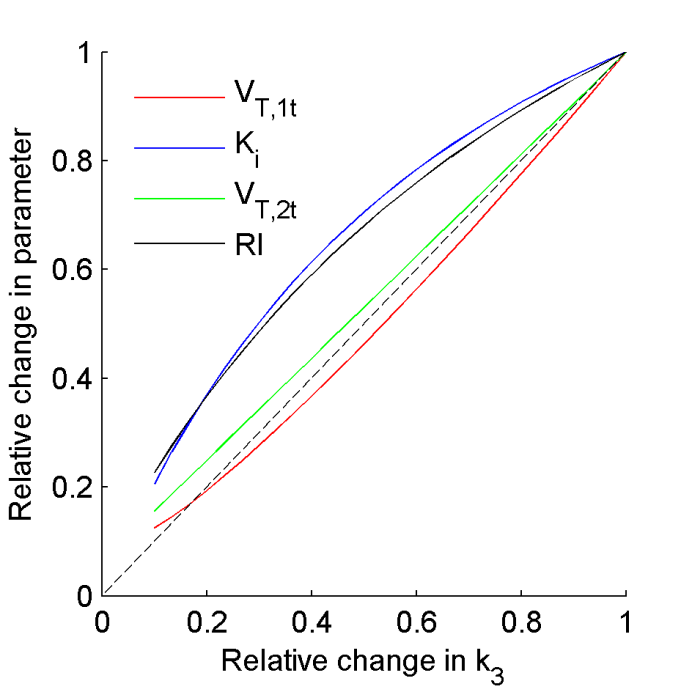

Supplement: Additional file 1: Figure S1. — Results of sensitivity analysis, showing relative change in k 3 (x-axis, representing transport through the norepinephrine transporter) and resulting relative changes in fitted outcome measures. Dashed line represents line of identity. [file 13550_2014_52_MOESM1_ESM.docx]
